# A rare immunological disease, caspase 8 deficiency: case report and literature review

**DOI:** 10.1186/s13223-023-00778-3

**Published:** 2023-04-10

**Authors:** Narges Bazgir, Azin Tahvildari, Zahra Chavoshzade, Mahnaz Jamee, Zahra Golchehre, Abdollah Karimi, Naghi Dara, Mazdak Fallahi, Mohammad Keramatipour, Arezou Karamzade, Samin Sharafian

**Affiliations:** 1 Student Research Committee, School of Medicine, Shahid Beheshti University of Medical Sciences, Tehran, Iran; 2 Immunology and Allergy Department, Mofid Children’s Hospital, Shahid Beheshti University of Medical Sciences, Tehran, Iran; 3 Pediatric Infectious Research Center, Mofid Children’s Hospital, Shahid Beheshti University of Medical Sciences, Tehran, Iran; 4 Pediatric Nephrology Research Center, Research Institute for Children’s Health, Shahid Beheshti University of Medical Sciences, Tehran, Iran; 5 Department of Medical Genetics, School of Medicine, Tehran University of Medical Sciences, Tehran, Iran; 6 Pediatric Gastroenterology, Hepatology and Nutrition Research Center, Research Institute for Children’s Health, Shahid Beheshti University of Medical Sciences, Tehran, Iran; 7 Watson Genetic Laboratory, North Kargar Street, Tehran, Iran

## Abstract

**Background:**

Caspase-8 is a molecule in the FAS pathway that initiates apoptosis. One of the rarest autoimmune lymphoproliferative syndromes is caspase-8 deficiency. Immunodeficiency, splenomegaly, and lymphadenopathy are the common symptoms of this condition.

**Case Presentation:**

A two-year-old boy entered this study with a fever of unknown origin (FUO) and dysentery. Moreover, he suffered from failure to thrive and was allergic to the cow's milk protein. His fever and dysentery did not respond to antibiotic therapy. The colonoscopy revealed diffuse ulcerations regions in the sigmoid along with skipped areas, mimicking Crohn's disease aphthous lesions. He represented very early-onset inflammatory bowel disease (IBD) and was diagnosed with the caspase-8 deficiency.

**Conclusion:**

There can be diarrhea or dysentery as the first or main symptoms of inborn errors of immunity (IEIs). The cause of diarrhea and dysentery in this case was early-onset IBD. One of the symptoms of IEIs such as caspase-8 deficiency is early-onset of IBD. Patients with early-onset had normal T cell count and low or normal immunoglobulin levels with insufficient immune response.

## Introduction

Caspase-8, encoded by the *CASP8* gene, initiates apoptosis’s extrinsic pathway. Caspase-8 is downstream of cell surface death receptors like FAS [1]. FAS receptors have extracellular, intracellular and transmembrane domains. Upon activation, the intracellular domain recruits FAS-associated death domain (FADD), which is made up of two domains including, the death domain (DD) and death effector domain (DED) [1, 2]. The DED heterodimer formation resulted in the recruitment of additional procaspase-8. The procaspase 8 interacts with the DED of the previously formed procaspase-8 [3].

Procaspase-8 proteins undergo autoproteolytic cleavage once their proteolytic domains homodimerize. Caspase-8 is ultimately activated as a result of the autoproteolytic cleavage [4].

Caspase-8 deficiency is an autosomal recessive subtype of the broad-spectrum autoimmune lymphoproliferative syndrome (ALPS) [5]. Embryonic lethality, abnormal heart muscle, and decreased hematopoietic precursors have all been observed in homozygous caspase-8 deficient mice [6]. Impairment of lymphocytic apoptosis in humans leads to accumulation of lymphocytes in the lymphatic organs. Splenomegaly and lymphadenopathy may ensue as a result [5, 7]. Immunodeficiency is another manifestation of caspase-8 disease since the enzyme is important for lymphocyte activation [1, 7]. Several treatment modalities are used for ALPS. Immunosuppressive agents like mycophenolate mofetil and sirolimus are used as treatments for this illness. A definite therapy for ALPS is hematopoietic stem cell transplantation (HSCT) [8].

Caspase-8 deficiency is a rare and often fatal immunological disorder, it is crucial to diagnose patients suffering from this disease in order to appropriately manage their symptoms..

The purpose of this study was to report a caspase-8 deficient patient and review other cases who have been reported in the literature.

## Material and methods

### Subject

**Fig. 1:**
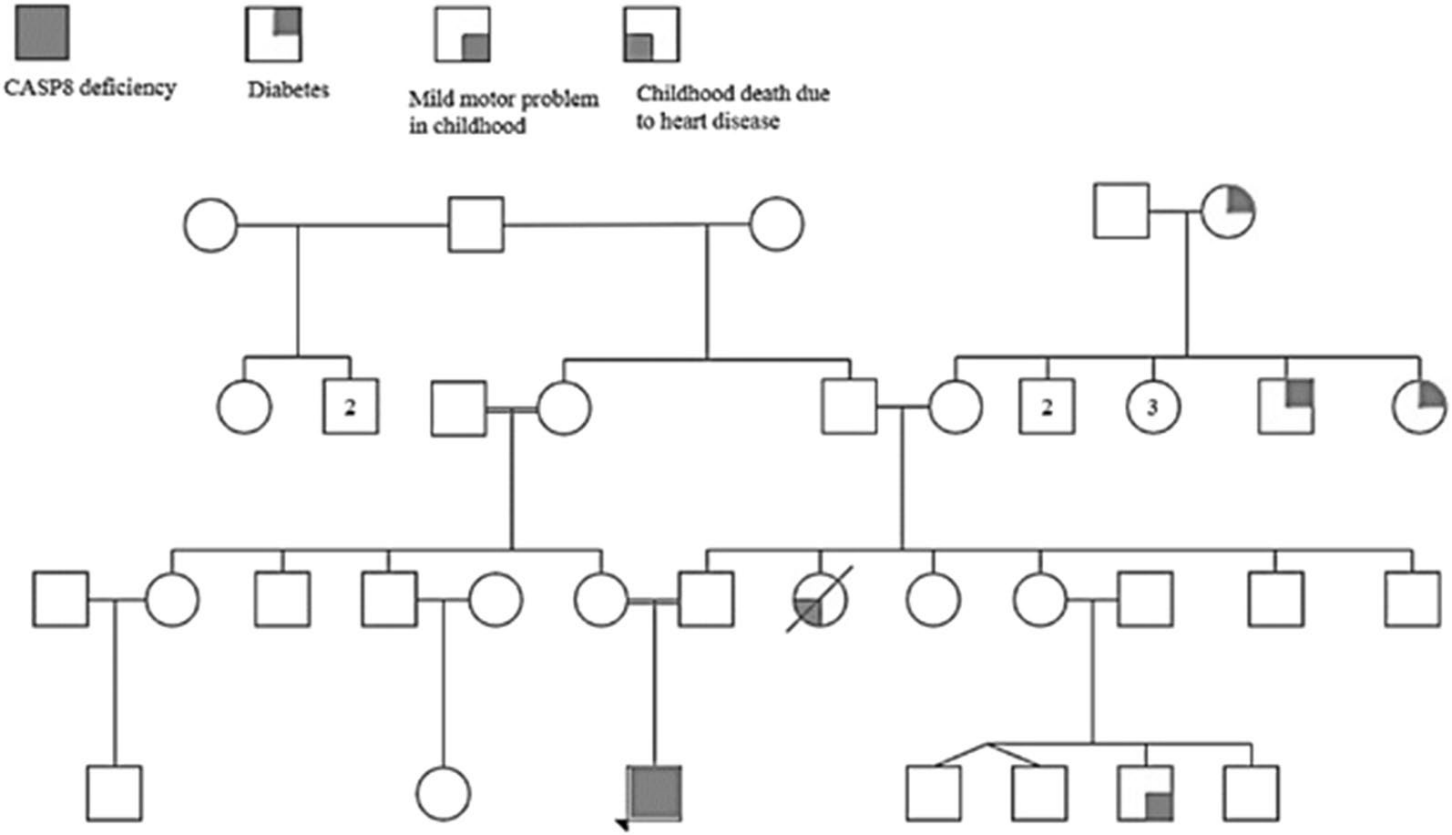
The pedigree of the proband and his family. The proband is represented by a filled square and other conditions in pedigree has been shown with other symbols

A two-year-old boy was hospitalized at Mofid Children's hospital with a history of prolonged fever and dysentery for 50 days. His parents were consanguineous, and he was the sole child (Fig. 1). He underwent physical and comprehensive paraclinical evaluations.

### Genetic analysis

Written informed consent was received from the parents of the patient. Afterward, blood samples were taken from the patient, his mother and father in the tubes containing 0.5 mmol ethylene diamine tetra acetic acid (EDTA). The ExgeneTM Blood SV DNA purification kit (GeneAll Biotechnology, Korea) was utilized to isolate the genomic DNA from whole blood according to the manufacturer's instructions [9].

The patient’s whole exome sequencing (WES) was performed by CeGaT GmbH (Tübingen, Germany) with an average coverage of roughly 100 × . Several public databases, including ANNOVAR [10], were used to annotate variants with an observed frequency of the alternative allele (OFA) of > 0.02. Subsequently, variants were filtered based on population allele frequency < 0.05 in the Exome Aggregation Consortium (ExAC [11]) Genome Aggregation Database (gnomAD [12]) and an in-house database of over 2000 Iranian exomes. Variants located in exonic regions and flanking ten base pairs (bp) were included for further analysis. In addition, synonymous variants located outside of splicing regions were excluded. Included variants were prioritized based on the correlation of the associated gene with the patient's phenotype. Multiple lines of in silico prediction tools, namely MutationTaster [13], Combined Annotation-Dependent Depletion (CADD [14]), and Deleterious Annotation of genetic variants using Neural Networks [15] PhyloP, PhastCons and GERP [16] were used to evaluate the impact of the identified variants. Furthermore, Human Gene Mutation Database (HGMD), ClinVar [17], and literature were searched to find previous reports of the variant. Finally, the considered variants were classified based on the American College of Medical Genetics and Genomics (ACMG) standards and guidelines for interpreting sequence variants [18].

Sanger sequencing was conducted using ABI 3500 Genetic Analyzer to validate the detected *CASP8* variant in the patient and confirm the carrier status of his parents.

## Results

### Case description

The patient was full-term at the time of vaginal delivery. He had a history of repeated hospitalizations for fever of unknown origin (FUO) and dysentery. Despite antibiotic therapy throughout, no remission had been observed. In addition to minor thalassemia, he had a cow's milk allergy, as well as multiple anal fissures that were surgically repaired. He was vaccinated according to the national vaccination protocol with no adverse reactions. Though underweight, he was neurologically and developmentally normal with a Z score of -3.31 (0.093 percentile). The physical examination revealed cervical lymphadenopathy, splenomegaly, and anal fissure. He underwent a complete workup to rule out infectious diseases. Table 1 shows the results of different para-clinical evaluations performed on the patient. Moreover, the results of flow cytometry and lymphocyte transformation tests are also shown in Table 1.

**Table 1:** The immunological and laboratory findings for the patient in this study

Diagnostic workup	Results	Normal range
WBC (*10^3^/μL)	9.3 (neutrophil = 33, lymph = 59)	5.5–15.5
Hb (g/dL)	6.6	10.5–14.5
PLT (*10^3^/μL)	810	150–450
BC	Negative	
UA	Normal	
S/E	Normal	
S/C	Normal	
CRP (mg/L)	75	< 10
Bone marrow PCR	Normal	
HIV Ab	0.2 (Negative)	< 1
PBS Malaria	Negative	
PBS Borrelia	Negative	
ESR (mm/hr)	31	< 10
Wright	Negative	
CMV PCR	Negative	
Fungi PCR	Negative	
TB PCR	Negative	
Mycobactrium PCR	Negative	
Anti Diphtheria Ab (IgG)	> 1 (Positive)	< 0.1: No response 0.1–1: Poor response >1: Normal response
Anti-Tetanus Ab (IgG)	3.3 (Positive)	< 0.1: No response 0.1–1: Poor response >1: Normal response
Bacteriology culture	Negative	
BACTEC	Negative	
Na (mEq/L)	132	135–145
K (mEq/L)	5.1	3.5–5
Sweat chloride test	Negative	0–40: Negative
Stool calprotectin	1012	50–200
BUN (mg/dL)	10.2	5–18
Cr (mg/dL)	1.5	0.5–1
Ph (mg/dL)	4.9	4–7
AST (U/L)	24	10–40
ALT (U/L)	14	10–40
ALP (U/L)	417	< 350
WBC (*10^3^/μL)	10	5.5–15.5
Hb (g/dL)	7.4	10.5–14.5
PLT (*10^3^/μL)	860	150–450
C-ANCA	Negative	
P-ANCA	Negative	
Anti-TTG	IgA:0.1 IgG:2.6	IgA:0–4 IgG:0–5
CD3 (%)	52 (decreased)	57–75%
CD4 (%)	10 (decreased)	28—47%
CD16 (%)	10 (normal)	3–15%
CD19 (%)	27 (normal)	14–33%
CD8 (%)	22 (normal)	18–35%
CD56 (%)	10.7 (normal)	3–15%
PHA	4.1	> 3
BCG	3.6	> 2.5
Candida	2.5	> = 2.5
NBT	100	> 95

*WBC* white blood cell, *Hb* hemoglobin, *PLT* platelet, *BC* blood culture, *UA* urinary analysis, *S/E* stool exam, *S/C* stool culture, *CRP* C-reactive protein, *HIV ab* HIV antibody, *PBS* peripheral blood smear, *ESR* erythrocyte sedimentation rate, *BUN* blood urine nitrogen, *Cr* creatinine, *Ph* phosphorus, *AST* aspartate transaminase, *ALT* alanine transaminase

**Fig. 2:**
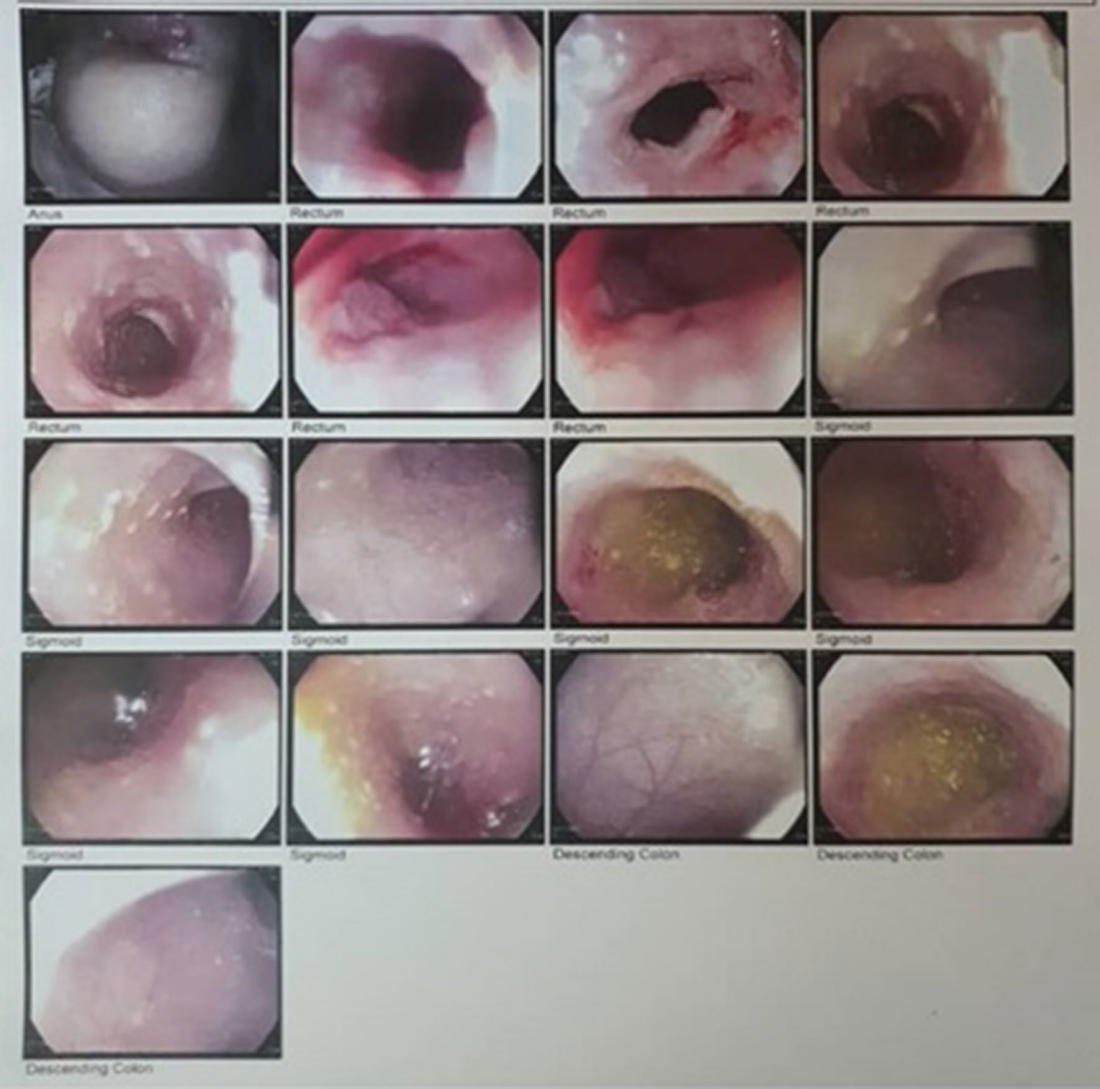
The colonoscopy of the patient. Rectum had mucosal erythema with diffuse ulcerative lesion and mucosal friability. Diffuse ulcerative lesions with skipped area mimic Crohn like aphthous lesion were evident in sigmoid. One deep ulcerative lesion with active bleeding in proximal descending colon was observed

Multiple polyploidy lesions with dispersed ulcers and hemorrhage were discovered during his colonoscopy. The diffuse ulceration region in the sigmoid with skipped areas mimics Crohn-like aphthous lesion (Fig. 2). A 1.5-cm-long stenotic area was seen 0.5 cm away from the surgical margin. As a result, he underwent an ileostomy and colostomy.

During endoscopy, reflux esophagitis grade A, gastritis erythematous, and nodularity were observed. His H. Pylori test result was negative. On superficial soft tissue sonography of the perianal region, there were no evidence of collection or abscess.

A hematology consultation ruled out malignancy and hemophagocytic lymphohistiocytosis (HLH). Bone survey and bone marrow biopsy were normal.

The symptoms of FUO, dysentery and FTT suggest he may have immunodeficiency. The results of his immunological tests revealed normal lymphocyte transformation tests (LTT) and decreases in CD3, CD4, and CD16 populations.

The results of immunological tests revealed normal lymphocyte transformation test (LTT) and decrease in CD3, CD4, and CD16 populations.

During his hospitalization, he received IVIG three times. He experienced frequent episodes of fever and diarrhea despite being given IVIG. He was discharged from the hospital with antibiotics, omeprazole, and sachet granule Mesalazine. A monthly IVIG was also administered to him.

### Genetic findings

A homozygous missense variant (c.1572G > T, p.Q524H) was detected in exon 9 of the *CASP8* gene (NM_001080125) out of a total of 55,356 variants (Table 2). This variant was not found in 1000G, ExAC, GnomAD, and the Iranian local database. Furthermore, in silico analysis predicted that this variant is deleterious (scores for CADD = 22.4, SIFT = 0, and PolyPhen = 0.944; MutationTaster predicts that as a disease causing variant) and affects a conserved amino acid. To the best of our knowledge, this variant has not yet been reported in constitutional (inherited) status.

**Table 2:** Genetic result of the patient

Gene/transcript	Variation location	Variant	Chromosome position (GRCh37)	Relationships	Zygosity	Variation classification^a^
*CASP8*, ENST00000358485.4 and NM_001080125	Exon 9	c.1572G > T, p.Q524H	Chr2:202,151,272	Patient	Homozygous	Likely Pathogenic
				Father	Heterozygous	
				Mother	Heterozygous	

^a^Based on American College of Genetics and Genomics (ACMG) standards and guidelines for the interpretation of sequence variants, 2015

**Fig. 3:**
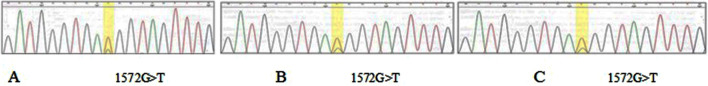
The chromatograph of the P8 mutation in caspase-8 gene. Illustration of the homozygosity in patient 8 (1572G > T) (**A**), the heterozygosity in the patient's father (**B**), the heterozygosity in the patient's mother (**C**)

Based on ACMG guidelines, this variant met PS3 (Well-established functional study, as mentioned above), PM2 (absent in population databases), and PP3 (computational evidence) criteria. This variant would therefore be categorized as likely pathogenic. Sanger sequencing confirmed the homozygosity of the case and the heterozygosity of his parents. Figure 3 displays the zygosity status of the patient and his parents.

The patient had a pathogenic variant in the HBB gene, considering the minor thalassemia status of the patient (NM_000518; c.93-21G > A).

## III. Literature review

### Clinical manifestations

Herein, we reviewed eight patients with caspase-8 deficiency. The first two early-onset patients in Table 3 (P1, P2) had lymphadenopathy and splenomegaly. They both showed sinopulmonary herpetic infections and did not respond well to immunization [19]. Although recurrent mucocutaneous herpetic infection is a common feature of caspase-8 deficiency [19], herpetic infection was not observed in the case of this study.

**Table 3:** The demograghic data, immunological and genetic findings of 8 patients with caspase-8 deficiency

Subject	Patient 1^a^ [19]		Patient 2^a^ [19]			Patient 3^b^ [20]		Patient 4^b^[20]	Patient 5 [21]	Patient 6 [21]	Patient 7 [21]	Patient 8
Age of onset (Year)	12y		11y			38y		37y	Whitin the first years of life	Within the first years of life	Within the first years of life	2y
Gender	Female		Male			Female		Male	NA	NA	NA	Male
First presentation	LAP and splenomegaly		LAP and splenomegaly			Acute shortness of breath		Complex neurological syndrome	Recurrent fever	Bloody diarrhea, recurrent infections and fever	Non-bloody diarrhea, recurrent infections	FUO, dysentery
Symptoms	Poor response to immunization, organomegaly, sinopulmonary and herpes simplex virus infection		Poor response to immunization, organomegaly, sinopulmonary and herpes simplex virus infection			Pneumonia, Pulmonary hypertension, Interstitial lung disease, high grade fever, pancytopenia, multiple necrotizing and non-necrotizing granulomas of lung		1 cm3 mass at the Meckel’s cave(necrotizing granuloma) aspiration pneumonia, bronchiectasis, organomegaly Multinodular lesions in liver and spleen, cranial nerve palsy/paresis	FTT, diarrhea, perianal disease, proctocolitis, refractory colitis increased susceptibility to bacterial and viral infections, multiple food allergy with mild peripheral eosinophilia and chronic eosinophilic infiltration antrum and duodenum Chronic eczema, pityriasis amiantacea, and hypothyroidism	FTT, diarrhea, perianal disease, proctocolitis, increased susceptibility to bacterial and viral infections, Refractory colitis	FTT, perianal disease, proctocolitis. increased susceptibility to bacterial and viral infections, intestinal obstruction, Refractory colitis	FUO, dysentry, FTT, lymphadenopathy, allergy to cow's milk protein, splenomegaly, Perianal fissures, Crohn's disease
Failure to thrive	+		+			−		−	+	+	+	+
Lymphadenopathy	+		+			−		−	+	−	−	−
Splenomegaly	+		+			−		+	+	−	−	+
Eczema	+		+			−		−	+	−	−	−
Reactive airway disease	+		+			−		−	−	−	−	−
Pneumonia	+		+			+		+	+	Not mentioned	+	−
HSV labialis	+		+			−		−	−	−	−	−
Chronic diarrhea	−		+			−		−	+	+	+	+
Food allergy	−		−			−		−	+	−	−	+ (bovine protein allergy)
Infections	sinopulmonary and herpes simplex virus infection		sinopulmonary and herpes simplex virus infection			Acute EBV infection, Nocardia asteroids meningoencephalitis, pneumonia		Aspiration pneumonia	Pneumonia, otitis media, conjuctivitis, purulent dermatitis, molluscum contagiosum, and local infections			
Cause of death							Nocardia asteroids meningoencephalitis	Progressive neurological and pulmonary complications	Alive	Septic complications	Alive	Alive
Genetic test		homozygous *CASP 8* mutation c.919C > T; p.R307W		homozygous *CASP 8* mutation c.919C > T; p.R307W			homozygous *CASP 8* mutation c.919C > T; p.R307W	homozygous *CASP 8* mutation c.919C > T; p.R307W	homozygous *CASP8* mutation c.836A>G, p.Q279R	homozygous *CASP8* mutation c.836A > G, p.Q279R	homozygous *CASP 8* mutation c.919C > T; p.R307W	homozygous *CASP8* mutation c.1572G > T p.Q524H
Immunoglobulin concentration		IgG: normal IgA: normal IgM: normal IgE: normal		IgG: low IgA: normal IgM: low IgE:normal			IgG: normal IgA: normal IgM: Low IgE: normal	IgG: normal IgA: normal IgM: Low IgE: normal	Not available	Not available	Not available	IgG: normal IgA: low IgM: normal
Total lymphocytes	1510 (normal)		2362 (normal)			990 (low)		3710 (high)	Not available	Not available	Not available	5487 (high)
CD4%	23.1 (low)		25 (low)			23.5 (low)		9.3 (low)	Not available	Not available	Not available	10
CD8%	49.6 (normal)		46.2 (normal)			65.6 (normal)		87.6 (high)	Not available	Not available	Not available	22
CD4/CD8 ratio	0.5 (low)		0.5 (low)			0.35 (low)		0.1 (low)	Not available	Not available	Not available	0.45 (normal)
B Lymphocytes	361		570			Not available		Not available	Not available	Not available	Not available	Not available

(Patient number 8 is the case presented in this study)

*FTT* failure to thrive, *LAP* lymphadenopathy, *FUO* fever of unknown origin, *NA* Not Available

^a^Patient number 1 and 2 were siblings

^b^Patients number 3 and 4 were siblings

The third case (P3) began to develop symptoms two months after giving birth to her baby. She experienced severe pulmonary involvement, a high fever, and pancytopenia. She underwent a lung transplant as a result of organ failure. The most incapacitating symptoms were recurring infections at different sites. She eventually died of meningoencephalitis caused by Nocardia. The complex neurological syndrome was the first manifestation and the cause of death in the fourth case (P4) [20].

The three other patients (P5, P6, P7) from unrelated consanguineous parents developed chronic diarrhea and failure to thrive and were eventually diagnosed with very early-onset IBD. Aside from having perianal disease, severe structuring, and fistulizing proctocolitis, they were prone to bacterial and viral infections [21]. Despite IBD being a frequent manifestation of caspase-8 deficiency, the mechanism is still unknown [21].

The clinical manifestations of caspase-8 deficiency can vary by age of onset, as shown in Table 3.

Multiple organ failure, organomegaly, and lymphocyte infiltrations are common symptoms of adult-onset disorders [20]. Furthermore, the nervous system was involved in all adult-onset cases [20]. In contrast, early-onset patients typically suffer from FTT, organomegaly, and infection. In all cases, the development was normal [5, 19, 21]. Hypersensitivity is common among individuals with early-onset disease, but none of the adult-onset patients exhibited with hypersensitivity [19]. In both early-onset and adult-onset patients, the only common symptom was recurrent infections.

Diarrhea and dysentery may be the first or main symptoms in patients with inborn errors of immunity [21]. The initial symptom in our case was a bloody stool, which was misdiagnosed as cow's milk allergy and infectious gastroenteritis, but elemental formula and antibiotics were ineffective. Consequently, a pediatric gastroenterologist suspected IBD. In light of this, it is relevant to note that very early-onset IBD (VEO-IBD) is an unusual condition, so these patients must be screened for inborn errors of immunity. IBD that appears under the age of 6 is called VEO-IBD [22]. In most cases of monogenic IBD, children younger than six years old are affected. The monogenic IBD is more prevalent among infants less than two years old, who are classified as infantile-onset IBD [22, 23]. The underlying causes of monogenic IBD are often PIDs, which indicates the importance of immune system dysregulation such as caspase-8 deficiency in VEO-IBD [22]. In our case as well as other reports, FTT, chronic diarrhea, eczema, other allergic disorders, lymphadenopathy, and splenomegaly were observed as the most common manifestations of early-onset caspase-8 deficiency.

### Laboratory findings

As shown in Table 3, our case had a higher number of lymphocytes than the two previous early-onset cases. Early-onset cases had CD4/CD8 ratio of around 0.5.

In both cases of late-onset disease, the immunoglobulin levels changed in a similar way. Low level of IgM was observed in both cases [20].

Serum immunoglobulins level could be in the normal range or low, but the humoral response was insufficient. Despite normal T-cell counts, pediatric patients had reverse CD4 + /CD8 + ratios.

### Genetic abnormalities

In eight patients with caspase-8 deficiency, three different homozygous variants including (NM_001080125): c.919C > T (p.R307W), c.836A > G (p.Q279R), and c.1572G > T (p.Q524H) were found.

### Potential effects of the different mutations on molecular mechanisms

Two mutations p.R307W and p.Q279R are located in the large catalytic p18 domain of caspase-8 [24] so they might affect the catalytic activity of the protein. It has been reported that the variant p.R307W causes the protein to lose its enzymatic properties, as well as reducing its stability [19].

The germline homozygous Q524H variant (they named this variant as the Q482H, based on NM_001228.4 transcript) detected in our patient has been previously reported in 7.53% acute myeloid leukemia (AML) cases with somatic status. They found that the Q524H variant hindered the dimerization of procaspase 8. Consequently, caspase-8 mediated apoptotic signaling is impaired, and chemotherapy resistance may eventually develop. Anti-apoptotic effect was functionally confirmed [25], and it is consistent with the underlying ALPS-IIB mechanism.

A number of patients have reportedly been impacted by ALPS-IIB. In a recent study, a patient with similar phenotypes to our patient was reported to have another homozygote missense variant (c.1358C > T;p.Pro453Leu)[26]. The authors found impaired cleavage of procaspase 8 and decreased apoptosis induced by FAS in effector memory T cells.

In conclusion, caspase-8 deficiency is a rare immunological disorder with various clinical presentations. It is recommended that patients with recurrent infections and atypical immunological symptoms be evaluated for this deficiency.

This manuscript presented the clinical manifestations of the eighth caspase-8 deficiency with IBD-like syndrome. We also reviewed the previous 7 caspase-8 deficiency cases and compared early-onset and adult-onset cases.

## Data Availability

Not applicable.
